# Factors Predicting Renal Function Outcome after Augmentation Cystoplasty

**DOI:** 10.1155/2017/3929352

**Published:** 2017-03-06

**Authors:** Shahbaz Mehmood, Raouf Seyam, Sadia Firdous, Waleed Mohammad Altaweel

**Affiliations:** ^1^Department of Urology, King Faisal Specialist Hospital & Research Centre, P.O. Box 3354, Riyadh 11211, Saudi Arabia; ^2^Fatima Jinnah Medical University, Lahore, Pakistan

## Abstract

We determined the cause of renal deterioration after augmentation cystoplasty (AC). Twenty-nine adult patients with refractory bladder dysfunction and who underwent ileocystoplasty from 2004 to 2015 were studied. Patients with a decline in glomerular filtration rate (GFR) after augmentation were reviewed. The primary outcome was to determine the factors that might lead to deterioration of estimated GFR. Median follow-up was 7.0 ± 2.6 years. Significant bladder capacity, end filling pressure, and bladder compliance were achieved from median 114 ± 53.6 to 342.1 ± 68.3 ml (*p* = .0001), 68.5 ± 19.9 to 28.2 ± 6.9 cm H_2_O (*p* = .0001), and 3.0 ± 2.1 to 12.8 ± 3.9 (*p* = .0001), respectively. Renal function remained stable and improved in 22 (76%) patients from median eGFR 135 ± 81.98 to 142.82 ± 94.4 ml/min/1.73 m^2^ (*p* = .160). Significant deterioration was found in 7 (24%) patients from median eGFR 68.25 ± 42 to 36.57 ± 35.33 (*p* = .001). The causes of renal deterioration were noncompliance to self-catheterization (2 patients), posterior urethral valve/dysplastic kidneys (2 patients), and reflux/infection (2 patients). On multivariate analysis, recurrent pyelonephritis (OR 3.87, *p* = 0.0155) and noncompliance (OR 30.78, *p* = 0.0156) were significant. We concluded that AC is not the cause of progression to end-stage renal disease in patients with renal insufficiency.

## 1. Introduction

Augmentation cystoplasty (AC) has traditionally been used in the management of small capacity, poorly compliant, or refractory overactive bladder. Severe bladder dysfunction has deleterious effects on the upper renal tract in terms of renal function deterioration in both native and transplanted kidneys [1]. High storage pressure because of bladder dysfunction can cause vesicoureteric reflux and subsequently impair renal function [2]. It has been estimated that almost 15% of end-stage renal disease (ESRD) cases were caused by lower urinary tract dysfunction.

Various congenital anomalies may result in small capacity, poor compliance, and high intravesical pressure, which threaten upper tract function. When these bladder conditions are refractory to conservative therapy, augmentation cystoplasty is required to preserve renal function. These diverse groups of conditions include posterior urethral valve (PUV), bladder and cloacal exstrophy, and epispadias and myelomeningocele [3–5].

In properly selected patients, augmentation cystoplasty is an excellent procedure that provides a safe and effective way of improving urinary storage. It provides long-term therapy in patients with refractory neurogenic bladder, but stomal problems continue to be a source of complication in the continent outflow channel [6]. Bladder emptying is almost universally impaired, and the patient must be prepared to perform lifelong intermittent catheterization. The patient and physician must recognize the need for surveillance to identify potential problems. Stones, metabolic and nutritional abnormalities, renal insufficiency, and malignancy are best treated through early recognition and prompt therapy [7].

Augmentation cystoplasty is used in an attempt to preserve and improve renal function. In spite of this, deterioration in renal function has been observed in 0 to 15% of patients after augmentation cystoplasty [8]. A few authors proposed that this is because of baseline renal function status. Some authors argued that augmentation cystoplasty may hasten the ESRD, proposing chronic renal insufficiency to be a relative contraindication for augmentation cystoplasty [9]. Others are of the opinion that augmentation cystoplasty does not appear to cause renal function deterioration in patients with chronic renal insufficiency [10]. In another large retrospective cohort study, it was found that that deterioration of renal function after augmentation cystoplasty was strongly associated with preoperative diagnosis of lower urinary tract dysfunction. It was further concluded that impairment of renal function is likely related to primary pathology rather than augmentation cystoplasty [11].

In view of the above controversy, we need to know the exact etiopathogenesis of renal deterioration after augmentation cystoplasty. The primary objective of our study was to determine factors that might predict the progression rate of renal insufficiency in patients with severe lower urinary tract dysfunction treated with AC.

## 2. Material and Method

After approval from the Office of Research Administration, adult patients with refractory bladder dysfunction who underwent augmentation ileocystoplasty by a single surgeon from 2004 to 2015 were retrospectively reviewed. All patients had refractory bladder dysfunction because of neurogenic and nonneurogenic etiology exhausted from all conservative and minimally invasive treatment.

### 2.1. Inclusion and Exclusion Criteria

Our inclusion criteria considered all adult patients over the age of 18 years who presented with bladder dysfunction. All patients with small capacity, noncompliant bladder with high or normal intravesical pressures proven by urodynamic testing were included in the study. Patients who failed maximum conservative therapy with strict (CIC), anticholinergic medication, and minimally invasive therapy in the form of intravesical botulinum toxin therapy were chosen for the study. We excluded patients with ESRD already on renal replacement therapy or those who underwent staged pretransplant augmentation cystoplasty because renal deterioration cannot be determined in these patients. We also excluded those patients who underwent ureterocystoplasty or augmentation done in another hospital. Patients in which only continent reconstruction were done, such as creation of Mitrofanoff, Monte, and bladder neck reconstruction without AC, were also excluded.

Patient demographics, diagnosis, surgical details, pre and postoperative urodynamic parameters, renal function, and postoperative complications were abstracted from patient medical records at our institution. Renal function was determined by calculating eGFR at baseline and last clinical follow-up using and age appropriate MDRD formula, such as GFR (ml/min/1.73 m^2^) = 186 × (Serum creatinine)^−1.154^  × (age)^−0.203^  × 0.742 (if female) × 1.212 (if black).

Patients were categorized according to the National Kidney Foundation criteria for Chronic Kidney Disease (CKD) on the basis of eGFR value in which stage 1 CKD is described as normal kidney function with eGFR > 90 ml/min/1.73 m^2^, stage 2 as kidney damage with a mildly decreased eGFR (60 to 89), stage 3 as moderately decreased eGFR (30 to 59), stage 4 as severely decreased eGFR (15 to 29), and stage 5 as kidney failure < 15 ml/min/1.73 m^2^ or requiring dialysis. All patients had some form of renal insult due to vesicoureteral reflux (VUR), pyelonephritis, scarring, or dysplastic kidneys fulfilling the CKD criteria.

The primary outcome of this study was to see renal deterioration from baseline and determine factors that might lead to renal insufficiency. Secondary outcome is to determine overall complication rates after augmentation.

### 2.2. Statistical Analysis

Data were analyzed by using Statistical Analysis Software (SAS) V. 9.3. Renal function and urodynamic parameters were presented as median ± standard deviation. Statistical analysis was performed using paired Student's *t*-test and Fisher's exact test. Univariate logistic regression analysis was performed to identify independent predictors of renal function deterioration after AC. Variables attaining *p* < .05 on univariate analysis or considered clinically relevant were included in the multivariate analysis. *p* value < .05 was considered significant.

## 3. Results

Of the 41 patients who had undergone lower tract reconstruction, 29 patients, 16 males (55%) and 13 females (45%), met inclusion and exclusion criteria. Median age at ileocystoplasty was 26 ± 08 (range 17–55) years. Patients were followed up for a median of 7.0 ± 2.6 (range 1–10) years after lower urinary tract reconstruction. Primary diagnosis for lower urinary tract dysfunction was neurogenic in 62% of patients (myelomeningocele: 10; spinal cord injury: 3; sacral agenesis: 3; postspinal surgery: 2) and nonneurogenic in 38% of patients (PUV: 2; bladder exstrophy: 6; nonneurogenic bladder: 2; schistosomiasis: 1). Preoperative urodynamic parameter revealed that 24 out of 29 patients had small capacity, low compliance, and high intravesical pressure. Five patients had small capacity, low compliance, and normal intravesical pressure. Baseline renal function was normal (eGFR > 90 ml/min/1.73 m^2^) in 18 of 29 patients (62%), 5 patients (17.24%) had CKD stage 2, 5 patients (17.24%) had CKD stage 3, and one patient had CKD stage 4. Bladder augmentation was done with ileum in all patients. Concomitant procedures included creation of continent outflow like Mitrofanoff in 14 patients, Monti neourethra in 2 patients, bilateral ureteric reimplantation in 2 patients, cecostomy button in 4 patients, bladder neck reconstruction in 4 patients, and pubovaginal sling in 3 patients. Two patients had nephrectomy for nonfunctioning kidney along with AC. The characteristics of study population are shown in Table 1.

Of the 18 patients with baseline normal renal function, 16 patients remained stable and improved their eGFR, except for 2 patients who progressed to CKD stage 2. One was having persistent vesicoureteral reflux and recurrent pyelonephritis confirmed on urine culture, voiding cystourethrogram (VCUG), and DMSA renal scan. The other patient was noncompliant to CIC and having high residual and recurrent urinary tract infection (UTI). Both patients remained stable after successful therapy on further follow-up. Categorization on the basis of eGFR is shown in Table 2.

Of the 5 patients with stage 2 CKD, 3 patients remained stable at stage 2. One patient progressed to stage 3, and the other progressed to ESRD. Both patients had a solitary functioning kidney, with contralateral nephrectomy done for nonfunctioning status along with bladder augmentation. Stage 3 patient had grade 5 reflux in the remaining kidney managed with ureteric reimplantation and strict CIC.

Renal function remained stable at stage 3 on further follow-up. One patient was incontinent and underwent bladder neck closure with Mitrofanoff created along with bladder augmentation. This patient experienced the complication of recurrent bladder and renal stones treated with PCNL, ESWL, and cystolithotripsy before progressing to ESRD.

Of the 5 patients with stage 3 CKD, one improved to stage 2, 2 remained stable at stage 3, and 2 deteriorated to ESRD and began hemodialysis. One of the 2 had a primary diagnosis of myelomeningocele and experienced persistent high intravesical pressure with recurrent symptomatic UTI. Another patient with posterior urethral valve fulgurated at childhood had dysplastic kidneys and renal scarring confirmed on renal ultrasound and DMSA renal scan. One patient had stage 4 CKD with fulgurated PUV and bilateral VUR as the primary diagnosis at the time of bladder augmentation and progressed to ESRD on follow-up. Overall, 22 patients (76%) remained stable and improved renal function (*p* = .160), and 07 patients (24%) deteriorated significantly from baseline (*p* = .0001) as shown in (Table 3).

We investigated certain factors (Table 4) in these progressive renal insufficiency patients after AC. Noncompliance to CIC, baseline creatinine, and preaugmentation renal insult, such as dysplastic kidneys, especially in patients with PUV, are some of the factors that might predict renal function deterioration after AC. Similarly persistent high intravesical pressure with VUR, recurrent symptomatic UTI, and metabolic disturbances are also factors that predict renal insufficiency on long-term follow-up after AC.

On univariate logistic regression analysis, factors like persistent vesicoureteral reflux (OR 13.333, 95% CI 1.65, 107.42, *p* value = .0150), recurrent pyelonephritis (OR = 125.97 95% CI = 6.81, 999.99, *p* value = .0012), and noncompliance to CIC (OR = 52.500 95% CI = 3.935, 700.52, *p* value = .0027) were found to be independent risks for renal function deterioration with respect to binary status, such as stable versus deteriorated renal function. When these significant factors were compared to each other on multivariate regression analysis, it was found that noncompliance to CIC was more significant (OR = 30.78, 95% CI = 1.913, 495.191, *p* value = .0156) than persistent vesicoureteral reflux (OR = 3.356, 95% CI = 0.193, 58.4, *p* value = .4064), and recurrent pyelonephritis was more significant (OR = 3.87, 95% CI = 2.089, 999.99, *p* value = .0155) than noncompliance to CIC (OR = 10.16, 95% CI = 0.314, 328.91, *p* value = .191). Although two patients had posterior urethral valves and both deteriorated, it was not found significant on univariate logistic regression analysis as shown in Table 5.

Regarding urodynamic study, patients achieved significant increase in bladder capacity with mean 342.1 ± 68.3 ml (*p* = .0001), decrease in end filling pressure 28.1 ± 6.9 cm H_2_O (*p* = .0001), and significant increase in bladder compliance 12.8 ± 3.9 ml/cm H_2_O (*p* = .0001) measured with formula: bladder compliance = Δ*v*/Δ*p*. This represents a 300% increase in bladder capacity (Table 6).

Regarding early complications, 2 patients who failed conservative management developed gross hematuria and underwent cystoscopy and coagulation of bleeding sites from augmented ileovesical junction. Three patients developed vesicocutaneous fistula; 2 were managed by keeping suprapubic and urethral catheter for one extra week. One patient was diverted with nephrostomy tubes and urethral and suprapubic catheter. Wound infection was found in 3 patients who were treated with IV antibiotics according to swab culture. Three patients were incontinent and two developed ileus managed conservatively (Table 7).

Three patients sustained bladder perforation. Although this is a dreadful complication, especially in neurogenic patients, we successfully managed all these cases by performing laparotomy and closure of the perforation. Perforation was found at ileovesical junction in all three cases. Mucus retention was also detected in all of the cases, which was primarily due to noncompliance of manual bladder irrigation and CIC. Two patients developed kidneys stones, and 5 patients got bladder stones, which were treated with ESWL, URS laser lithotripsy, and cystolitholapaxy, respectively. Out of 5 patients who complained of persistence of incontinence, 3 patients were managed with bladder neck reconstruction, with macroplastique injection at bladder neck, and 2 patients were managed successfully with anticholinergic medications. We did not find a single patient with bladder malignancy in our series.

Asymptomatic bacteriuria was found in most of the patients as >90% of our patients were doing CIC. Asymptomatic bacteriuria was found in 57% (15/29) of patients, and febrile UTI was found in 17% (05/29) of patients which were managed with oral or intravenous antibiotics according to culture and sensitivity and hemodynamic status of the patients.

## 4. Discussion

The primary objective of our study was to assess long-term renal function and to determine factors that might lead to deterioration of renal function after AC at our institution. The therapeutic goal of AC is to create low-pressure storage, large capacity, and a continent urinary reservoir. Decreased compliance and high pressure storage put the upper tract at risk for renal deterioration. Wang et al. [12] presented the deleterious effect of high detrusor leak point pressure on the upper urinary tract by calculating urodynamic risk score including a detrusor leak point pressure (DLPP) >40 cm H_2_O, bladder compliance of <9 mL/cm H_2_O, and evidence of an acontractile detrusor, in children with neurogenic lower urinary tract dysfunction. They found these three factors to be the main risk factors for upper tract dilatation and subsequent renal damage. None of them had reflux when DLPP was <40 cm H_2_O.

There is some controversy among authors on the role of AC in the preservation of renal function. A few studies have addressed this important issue [13, 14]. All of these showed good preservation of renal function after AC. Significant renal insufficiency is a more controversial relative contraindication. Few studies have addressed this issue in detail. Küss et al. [15] found that augmentation of patients with a creatinine clearance of >15 mL/min/1.73 m^2^ was associated with a 44% deterioration of renal function in one series while only 4.1% of renal deterioration was found when clearance was >40 ml/min/1.73 m^2^ [16]. They attested that this renal impairment may result in an inability to cope with the metabolic complication of AC.

In our series, AC stabilized and improved renal function in (*n* = 22) 76% of the patients, and 24% of the patients deteriorated to various stages. We found certain factors that can stabilize deteriorated renal function when corrected promptly. Fontaine et al. [17] achieved similar results. They observed that results of 10 years of follow-up study of AC in 53 patients showed that 19% of the patients experienced renal function deterioration expressed by a decrease in GFR of more than 20%. The most common reason for renal deterioration in these patients was chronic retention or infection because of inadequate catheterization due to poor compliance.

In younger age groups, the persistence of VUR after AC and its associated febrile UTI can impair renal function by recurrent pyelonephritis and renal scarring. Two of the patients in our series were having persistent VUR in their solitary functioning kidney. One of them stabilized after ureteric reimplantation. Soygur et al. found the necessity of ureteric reimplant after AC and observed that renal scarring and febrile UTI caused by VUR can impair renal function [18]. AC increases bladder compliance, by lowering intravesical pressure during the urinary storage phase; so in most cases, reflux improves after AC, making reimplantation unnecessary [19]. In our series asymptomatic bacteriuria was 57% (*n* = 15/29) and febrile UTI were found in 17% of our patients. Febrile UTI patients were treated with either IV or oral antibiotics according to sensitivity and patient's hemodynamic status. Greenwell et al. [20] found the same frequency of asymptomatic bacteriuria of 75% and troublesome febrile UTI of 20% in patients on CIC with AC.

More than 90% of our patients were on CIC. Three of our deteriorated patients were noncompliant to CIC associated with high residual and recurrent febrile UTI and pyelonephritis. One progressed to CKD 2 from normal eGFR > 90 ml/min/1.73 m^2^ and was stabilized at CKD 2 when CIC was enforced, and the other two progressed to ESRD from CKD 2 and CKD 3. These last two patients had other risk factors, such as solitary kidney complicated with bladder and renal stones, along with noncompliance to CIC. Noncompliance or inability to perform CIC is a relative contraindication to AC. Intermittent self-catheterization is simple, safe, and effective but underused procedure mandatory in neuropathic bladder, postoperative retention, and following bladder reconstruction like AC [21]. Intermittent catheterization preserves the upper urinary tract by eliminating residual urine, decreasing intravesical pressure, and reducing urinary infection. CIC is excellent in the preservation of the upper urinary tract. Noncompliance to CIC deteriorates renal function with reported range of 0 to 14% in various series [22]. Dik et al. stated that early start of therapy in the form of CIC and anticholinergic medications preserve renal function in patients with neuropathic bladder dysfunction and deteriorate in patients who were noncompliant to CIC [23].

We failed to achieve low intravesical pressure and higher compliance bladder and were unable to relieve lower urinary tract symptoms in one patient (3.44%) with myelomeningocele in our series. Unfortunately, that patient progressed to ESRD and required revision AC surgery before proceeding to renal transplantation. Failure of AC to relieve lower urinary tract symptoms and urodynamic parameter required to preserve upper tract has been reported in 5 to 42% of the patients in various studies [24, 25]. The success rate is lower in idiopathic detrusor overactivity patients (53–58%) [26] when compared to the higher success rate (almost 92%) reported in neuropathic patients [27].

A primary diagnosis of PUV is a nonmodifiable factor that has the worst prognosis in terms of renal function deterioration. PUV can lead to deleterious effects on bladder and renal function in long-term follow-up [28]. Renal function in PUV patients depends upon various well-known factors like age at presentation, GFR, renal dysplasia, VUR, renal scarring, extent of bladder dysfunction, and UTI. As many as 25 to 60% of PUV patients may have significant renal function impairment despite efforts made to treat these patients in long-term follow-up [29].

Two patients with primary diagnosis of PUV, one with CKD stage 3 and other with stage 4, progressed to ESRD after AC. Both patients had renal dysplasia and scarring on USG and DMSA scans. In contrast, congenital renal deterioration is rare in exstrophy patients before surgical reconstruction. Therefore, renal dysplasia, scarring, or intrauterine nephropathy can be ruled out as a cause of subsequent renal function deterioration [30]. One out of 6 bladder exstrophy patients deteriorated from normal renal function to CKD 2 due to noncompliance to CIC and recurrent UTI. We managed this patient with antibiotics, reeducated and reenforced to do CIC. This patient stabilized at CKD 2 on further follow-up.

These data show that overall AC stabilized and improved renal function in 76% of patients, and 24% deteriorated from baseline. We found certain remedial and nonmodifiable factors that lead to deterioration of renal function. Out of 7 deteriorated patients, 2 had primary PUV diagnosis and had inherent disease that lead to ESRD. All other factors like noncompliance to CIC, persistent VUR, recurrent pyelonephritis, and high pressure reservoir are modifiable when corrected lead to stabilization of kidney functions. Our data showed that close follow-up is essential in deteriorated patients to search for early modifiable factors. Although baseline renal function and primary diagnosis are significant, we need to correct modifiable factors to stabilize and at least prolong the time to develop ESRD after AC.

The strengths of our study are its long-term follow-up and detailed renal function analysis using eGFR. We admit methodologic constraints of retrospective analysis and low number of patients as main limitation of this study. The result of this study might not be generalized because of small sample size and heterogeneous group of the patients. Missing data and selection bias are inherent limitation of any retrospective study. Furthermore, our investigation is not without limitation. Serum creatinine was used to calculate eGFR which is only reliable in individuals in a steady state. The most important factors affecting serum creatinine are hydration status, exposure to contrast dye, variation in diet, muscle mass, and urinary tract infection. We did not control any of these factors in our retrospective analysis. However, the widespread clinical use of creatinine measurement makes it frequently available for analysis.

## 5. Conclusion

There is no evidence that AC causes renal damage. Close follow-up is necessary in patients with deteriorated renal function to search for remedial and modifiable factors that lead to renal function deterioration.

## Figures and Tables

**Table 1:** Demographic data.

Total number of patients	29
Gender	
Male	16
Female	13
Median age (years)	26 ± 08 (17–55)
Median follow-up (years)	7.0 ± 2.6 (range 1–10)
Pre-op renal function	
Normal eGFR	18
CKD stage 2	05
CKD stage 3	05
CKD stage 4	01
Pre-op diagnosis	
(1) Neurogenic	18
MMC	10
SCI	03
SA	03
Postspinal surgery	02
(2) Nonneurogenic	11
PUV	02
BE	06
NNB	02
Schistosomiasis	01
Urodynamic findings	
Small capacity, low compliance, and high pressure	24
Small capacity, low compliance, and normal pressure	05
Additional procedures	
Mitrofanoff	14
Monti	02
Pubovaginal sling	03
B/L ureteric reimplantation	02
Bladder neck reconstruction	04
Cecostomy button	04

PUV = posterior urethral valve; MMC = myelomeningocele; BE = bladder exstrophy; SA = sacral agenesis; SCI = spinal cord injury; NNG = nonneurogenic neurogenic bladder.

**Table 2:** Categorization of patients on the basis of eGFR.

Groups	Preoperative eGFR	Postoperative eGFR	*p* value
eGFR > 90 ml/min/1.73 m^2^ (*n* = 18)	160.4 ± 73.3	164.4 ± 91	.567
CKD 2 (*n* = 05)	70.4 ± 10.8	56.6 ± 28.2	.636
CKD 3 (*n* = 5)	40.2 ± 7.9	29.0 ± 21.4	.148
CKD 4 (*n* = 01)	19.0	10.0	n/a

**Table 3:** Overall stabilized and deteriorated renal function patients.

Category	Number of patients	Preoperative eGFR (ml/min/1.73 m^2^)	Postoperative eGFR (ml/min/1.73 m^2^)	*p* value
Stabilized/improved renal function	22 (76%)	135.50 ± 81.98	142.82 ± 94.45	.160
Deteriorated renal function	07 (24%)	68.29 ± 42.01	36.57 ± 35.33	.001

**Table 4:** Factors predicting renal deterioration.

S. number	Diagnosis	Pre-op Cr.	Post-op Cr.	Pre-op eGFR	Post-op eGFR	Probable cause of renal deterioration
1	MMC	73	98	120	85 (CKD 2)	Persistent VUR & recurrent UTI

2	BE	56	79	122	82 (CKD 2)	Noncompliance to CIC, recurrent pyelonephritis

3	MMC	102	157	83 (CKD 2)	50 (CKD 3)	Solitary left kidney with grade 4 reflux treated with ureteric reimplant, stabilized at CKD 3

4	MMC	120	612	66 (CKD 2)	10 (CKD 5)	Solitary kidneys with bladder neck closure and Mitrofanoff created having bladder & kidneys stone, noncompliant to CIC

5	MMC	250	426	30 (CKD 3)	6 (CKD 5)	Persistent high intravesical pressure, recurrent pyelonephritis, incontinent

6	PUV	200	510	38 (CKD 3)	13 (CKD 5)	Dysplastic & scarred kidneys

7	PUV(B/L VUR)	280	504	19 (CKD 4)	10 (CKD 5)	Dysplastic & scarred kidneys

**Table 5:** Predictive factors for renal function deterioration on univariate logistic and multivariate regression analysis.

Serial number	Predictive factors	Univariate analysis		Multivariate analysis	
		OR (95% CI)	*p* value	OR (95% CI)	*p* value
1	Persistent vesicoureteral reflux	13.333 (1.65, 107.42)	.0150	3.356 (0.193, 58.4)	.4064
2	Recurrent pyelonephritis	125.97 (6.81, 999.99)	.0012	3.87 (2.089, 999.99)	.0155
3	Solitary kidney with reflux	999.99 (0.001, 999.99)	.963		
4	Noncompliance to CIC	52.500 (3.935, 700.52)	.0027	30.78 (1.913, 495.191)	.0156
5	High pressure reservoir	13.556 (0.001, 999.99)	.963	—	—
6	Posterior urethral valves (PUV) with scarring and dysplasia	13.55 (0.001, 999.99)	.963	—	—
7	Renal/bladder stone/recurrent pyelonephritis	0.567 (0.055, 5.88)	.634	—	—
8	Solitary kidney without reflux	0.001 (.001)	.9831	—	—

**Table 6:** Pre- and postoperative urodynamic parameters.

Variables	Preoperative mean ± SD (range)	Preoperative mean ± SD (range)	*p* value
Mean capacity (ml)	114 ± 53.6 (40–270)	342.1 ± 68.3 (220–520)	.0001
Mean end filling pressure (cm H_2_O)	68.5 ± 19.9 (34–98)	28.2 ± 6.9 (18–45)	.0001
Compliance (ml/cm H_2_O)	3.0 ± 2.1 (.3 to 9.2)	12.8 ± 3.9 (7.1–21.6)	.0001

**Table 7:** Early and late complications.

	Number
Early complications	
Gross hematuria	2
Vesicocutaneous fistula	3
Incontinence	3
Ileus	2
Wound infection	3
Late complications	
Bladder perforation	03
Mucus retention	03
Bladder & kidney stones	05
Incontinence	05
Renal deterioration	07
Febrile UTI	07
Malignancy	Nil
